# Mental health services in the prisons of Bangladesh

**DOI:** 10.1192/bji.2021.34

**Published:** 2021-11

**Authors:** Al Aditya Khan, Howard Ryland, Tayeem Pathan, Helal Uddin Ahmed, Amir Hussain, Andrew Forrester

**Affiliations:** 1 FRCPsych, Consultant Forensic Psychiatrist, Oxleas NHS Foundation Trust, London, UK. Email: aladitya.khan@nhs.net; 2 MRCPsych, NIHR Doctoral Research Fellow, Department of Psychiatry, University of Oxford, Oxford, UK; 3 MRCPsych, Consultant Psychiatrist, Surrey and Border NHS Foundation Trust, Farnham, UK; 4 MD (Psychiatry), Associate Professor, Child, Adolescent and Family Psychiatry, National Institute of Mental Health, Dhaka, Bangladesh; 5 MPhil, General Member, Bangladesh Clinical Psychology Society, Dhaka, Bangladesh; 6 MD (Res), Professor of Forensic Psychiatry, Department of Psychological Medicine and Clinical Neurosciences, University of Cardiff, Cardiff, UK

**Keywords:** Bangladesh, prison, psychiatry and law, forensic mental health services, low- and middle-income countries

## Abstract

In this narrative review we consider what is known about mental health conditions in the prison system in Bangladesh and describe the current provision of mental health services for prisoners with mental health needs. We contextualise this within the literature on mental health conditions in correctional settings in the wider sub-continental region and low- and middle-income countries (LMICs) more broadly. We augment findings from the literature with information from unstructured interviews with local experts, and offer recommendations for research, policy and practice.

## Mental health in Bangladesh

Bangladesh is a country of 170 million people located in Southern Asia. It has one of the highest population densities of any country, with an average of over 1000 people per square kilometre. The per capita gross domestic product (GDP) is US$1855, placing it in the lower-middle-income category according to the World Bank. By comparison, per capita GDP is US$2100 in India and US$1285 in Pakistan.^1^

Mental disorders are common in Bangladesh, with a reported prevalence of 6.5−31.0% among adults and 13.4−22.9% among children.^2^ The overall prevalence of schizophrenia is 1.0% and major depressive disorder 6.7%.^3^ Although there is some awareness about the existence of mental disorders within the community, mental healthcare is not a priority. There is also often a negative attitude towards individuals affected by mental illness.

It is estimated that there are 0.53 physicians per 1000 population, with only around 220 psychiatrists and 50 clinical psychologists in the whole country. Overall, there are reported to be 0.49 mental health workers per 100 000 population. Bangladesh spends 1% of its GDP on health, of which 0.44% is spent on mental health. Insurance plans may not cover mental illness.^4,5^

In 2018, a new Mental Health Act was instituted in Bangladesh, replacing the Lunacy Act, which was more than a century old. This is a new Act and our understanding is that it is still to be fully implemented and the finer details will have to be clarified with time. It is generally viewed as an important step forward, but it has also been criticised for not going far enough in protecting the human rights of those with mental illness. Section 15 of the new Act allows for the admission of mentally disordered defendants or sentenced mentally disordered offenders under the direction of a magistrate, through the use of a ‘reception order’. No further details are specified in the Act. The Act does not specify or take into consideration any variation of provision for offenders at different stages of their criminal justice proceedings. The inclusion of provisions on criminal liability for providing false mental illness certificates has also raised concern. If mental health professionals are proven to have deliberately provided a false certificate, then they may face prosecution, resulting in a fine or imprisonment. Critics fear that this could have the unintended consequence of reducing access to care, as practitioners may avoid mental health consultations for fear of being asked to provide certificates, thus exposing themselves to potential legal action.^6^ We understand that the Prison Act is currently being revised to increase the emphasis on rehabilitation.

## The prison system in Bangladesh

According to the Department of Prisons,^7^ Bangladesh had 13 central jails and 55 district jails, holding a total of 73 177 prisoners, in 2016. This equates to 45 prisoners per 100 000, a similar level to Pakistan and India, and one of the lowest incarceration rates in the world. However, prison occupancy was 204% of capacity, indicating considerable overcrowding. In total, 3.9% of the prison population is female. There is one women-only prison in the country, located near Dhaka, and rest of the prisons have separate accommodation for men and women. Almost three-quarters of all prisoners (73.8%) were on remand, one of the highest ratios in the world, with many spending years awaiting trial. Of those convicted, 4905 prisoners had received a life sentence and 1204 were subject to the death sentence. Only 385 prisoners were considered ‘mentally challenged’ prisoners, with 7667 classified as ‘addicted’, although this is likely to be a considerable underestimate. There are no data on the number of prisoners who might be suitable for transfer to psychiatric hospital.

The goal of the five-year plan, 2016–2020 was to ‘Ensure healthy and safe custody of the prisoners through providing rehabilitation training, counselling and after release follow up’.^7^ A total of 173 prisoners died in 2016, representing a mortality rate of 2.43%, compared with 5.3% in the general Bangladeshi population and similar to the mortality rate among prisoners in India. Only 15 ‘unnatural’ deaths were recorded between 2002 and 2016, although further details are not publicly available. ‘Drug-related’ disease prevalence was recorded for 4596 prisoners, although no data were presented for mental illness. There was authorisation for one psychologist post in the whole prison estate, but this was vacant in 2016.^7^

## Mental health in prisons in Bangladesh

We did not identify any formal studies of the prevalence of mental illness in prisons in Bangladesh. However, a systematic review of the prevalence in LMICs included several studies from India. Pooled estimates for the total sample of 14 527 prisoners in 13 LMICs suggest rates of non-affective psychosis around 16 times that of the general population, substance misuse and major depression 6 times as common and alcohol misuse twice as common. The authors conclude that these rates are likely to reflect unmet need, requiring specialist solutions tailored to resource-limited settings.^8^ The high prevalence of mental illness identified in this review parallels findings from high-income countries, suggesting that this is a global phenomenon.^9^

Given the lack of published information, two of us (A.A.K. and T.P.) conducted telephone interviews with seven senior psychiatrists and a clinical psychologist in Bangladesh, identified through our professional networks. The interviewees were purposively selected to provide an accurate and comprehensive description of the current state of mental health services and practices in the country. All of the clinicians were based in leading national and regional mental health institutions that regularly offer assessment and treatment for mentally disordered offenders. Six of the psychiatrists worked for the government psychiatric service, one for a private medical university, and the clinical psychologist for a third-sector organisation. Five interviewees were based in the capital Dhaka and two in other districts. One psychiatrist (H.U.A.) and one psychologist (A.H.) interviewed are also authors of this review. Semi-structured interviews were used to determine the clinicians’ knowledge and perception of mental health need in prisons and the provision of mental healthcare in prisons by resident professionals, from visiting psychiatrists and other specialists and following transfer to specialist hospitals. Additionally, interviewees were asked to add further information that they felt was relevant.

Interviewees described significant psychiatric morbidity in the prison population in Bangladesh, with no formal psychiatric services within the prison to meet those needs. The prison medical workforce has limited training in mental health. Prison medical officers, who have bachelor's level general medical training, can assess and refer individuals with serious psychiatric symptoms. Patients can access free essential psychotropic drugs, including first-generation antipsychotics, lithium, valproic acid, benzodiazepines and amitriptyline. A wider range of drugs are available but must be purchased by patients themselves. Bangladesh does not have a specific mental health nursing qualification, but there are medical assistants trained as paramedics. The clinical psychologist interviewed confirmed that there are no psychologists currently working in prisons.

Third-sector organisations operate within prisons to provide support with training and counselling. One example is Dhaka Ahsania Mission (DAM), which provides group counselling on drug addiction and life skills development for prisoners through a project with the German non-governmental organisation Gesellschaft für Internationale Zusammenarbeit (GIZ). The project works closely with the Ministry of Home Affairs, the Ministry of Law, Justice and Parliamentary Affairs and the prison directorate to develop prison staff's knowledge and skills around substance misuse and mental health issues. It also provides support and follow-up after release for employment and abstinence from drugs. DAM recently provided staff training on COVID-19 preparedness in all 68 Bangladeshi prisons and basic mental health training covering stress management, anger management and mindfulness to all 13 central jails.

At present, there is an absence of systematic mental health screening in prisons. If a mental health opinion is needed, usually in cases of severe and acute illness, individuals are referred to the nearby psychiatric hospital. Prisons in Dhaka refer inmates to the National Institute of Mental Health Hospital. In other parts of the country, there are similar arrangements for assessment and treatment, as all teaching hospitals have a psychiatry department. Where there is no local teaching hospital, mentally disordered prisoners are transferred to a neighbouring district to access services. The police in Dhaka also have their own general hospital where people with physical or mental health problems can be admitted. Substance misuse services run their own hospital and provide opinions to prisons or courts, if requested.

Prisoners are escorted to assessments by two police officers at all times, as psychiatrists do not visit prison to assess inmates. Commonly, a medical board, consisting of psychiatrists and other doctors, will evaluate prisoners before formulating an opinion on whether an individual has major or minor psychiatric disorder. Treatment can then be provided as an out-patient or in-patient if needed. In principle, prisoners can be admitted for evaluation at any point on the criminal justice pathway, although in practice admissions are brief and only for acute cases. Once treated or stabilised on medication, they are returned to prison, with provision for prescriptions to be continued in prison. On release from prison there is also provision for referral to hospital or an out-patient service, but there is no probation service and consequently no provision for community sentences. The arrangements for the assessment and treatment of mentally disordered offenders are similar for both male and female prisoners.

Courts can refer defendants directly to local psychiatric institutions, if there are concerns about the presence of mental illness. Sentencing is usually carried out at the same hearing once guilt is established, so there is rarely the opportunity to get psychiatric reports. There is no provision for psychiatric evaluation in all capital cases, although a judge may waive the death penalty for acutely psychotic defendants, based on psychiatric evidence.

## Recommendations for future research

Given that the prevalence of mental disorders among prisoners is much higher than in the general population in other comparable LMICs, it is likely that these are also common in Bangladeshi prisons.^8^ Mental health provision for prisoners, however, appears to be very limited. Overcrowding, the high proportion of remand prisoners and high levels of mental illness in the community may all compound the situation. We recommend two avenues of initial enquiry to better understand any mental health treatment gap and guide service development.

Firstly, a formal survey of professionals working in prison, and of mental health professionals with links to prisons, using a structured or semi-structured questionnaire could be carried out to better understand the current level of service provision, training requirements and perceived need. Secondly, an epidemiological study is urgently needed to establish the prevalence of mental illness in prisons in Bangladesh. This should use screening instruments that are both culturally appropriate and validated for use in correctional settings.^10^ Once the level of need has been properly established, research and evaluations focusing on service development and interventions would be important.

## Recommendations for policy and practice

Authorities in Bangladesh may wish to consider cost-effective, scalable solutions to enhance the provision for their prisoners with mental illness. These could be adapted from successful interventions used in other countries, especially similar LMICs, or developed *de novo* to fit the needs of the local population. This could draw on innovative technologies, such as digital approaches to delivering mental health interventions.^11^ Interventions designed for use in resource-limited settings, such as psychological first aid and the World Health Organization's Mental Health Gap Action Programme (mhGAP), could be modified for use in the prison context. Raising awareness of mental illness and providing education on its identification, prevention and treatment could be targeted to both prison residents and staff. High levels of mental illness in the general population and limited community mental health services mean that imprisonment could be an opportunity to treat people who might otherwise not receive any support. A health economic evaluation could determine whether such interventions are ultimately cost-saving. The Department of Prisons could consider moving responsibility for mental healthcare to the health service. Improvements should ensure that the human rights of prisoners with mental illness are adequately protected. This may require adjustments to current mental health and criminal legislation to meet relevant international standards, such as those of the World Psychiatric Association and the United Nations’ 'Nelson Mandela Rules'.^12,13^

## Data Availability

Data availability is not applicable to this article as no new data were created or analysed in this study.
